# Indirect Selection on Flower Color in *Silene littorea*

**DOI:** 10.3389/fpls.2020.588383

**Published:** 2020-12-23

**Authors:** Nancy L. Rodríguez-Castañeda, Pedro L. Ortiz, Montserrat Arista, Eduardo Narbona, Mª Luisa Buide

**Affiliations:** ^1^Departamento de Biología Vegetal y Ecología, Facultad de Biología, Universidad de Sevilla, Seville, Spain; ^2^Departamento de Biología Molecular e Ingeniería Bioquímica, Universidad Pablo de Olavide, Seville, Spain

**Keywords:** calyx color, corolla color, floral display, florivory, male and female fitness, path analysis, phenotypic selection

## Abstract

Flower color, as other floral traits, may suffer conflicting selective pressures mediated by both mutualists and antagonists. The maintenance of intraspecific flower color variability has been usually explained as a result of direct selection by biotic agents. However, flower color might also be under indirect selection through correlated traits, since correlations among flower traits are frequent. In this study, we aimed to find out how flower color variability is maintained in two nearby populations of *Silene littorea* that consistently differ in the proportions of white-flowered plants. To do that, we assessed natural selection on floral color and correlated traits by means of phenotypic selection analysis and path analysis. Strong directional selection on floral display and flower production was found in both populations through either male or female fitness. Flower color had a negative indirect effect on the total male and female fitness in Melide population, as plants with lighter corollas produced more flowers. In contrast, in Barra population, plants with darker corollas produced more flowers and have darker calices, which in turn were selected. Our results suggest that the prevalence of white-flowered plants in Melide and pink-flowered plants in Barra is a result of indirect selection through correlated flower traits and not a result of direct selection of either pollinators or herbivores on color.

## Introduction

Biotic and abiotic agents of selection may affect floral traits and, thereby, plants fitness. Among the biotic agents, pollinators have been considered the most important (Darwin, 1862; Schiestl and Johnson, 2013; Souto-Vilarós et al., 2018; Ramos and Schiestl, 2019) because, given their primary role in transferring pollen between plants (Barrett and Harder, 1992), they have a strong and direct impact on plant fitness (Waser and Price, 1981; Waser et al., 1996; Schemske and Bradshaw, 1999). However, some floral traits can be signals not only to pollinators but also to herbivores (e.g., Raguso, 2008) that directly or indirectly damage reproductive tissues, reducing plant reproductive success (Gómez, 2003; Irwin et al., 2003). In this way, the potential selection exerted by pollinators could be altered by herbivores, changing the strength and the direction of that selection (Strauss, 1997; Hambäck, 2001; Herrera et al., 2002; Irwin et al., 2003; Irwin and Strauss, 2005; Ågren, 2019; Ramos and Schiestl, 2019). Thus, some floral traits suffer conflicting selective pressures mediated by both mutualists and antagonists (but see Veiga et al., 2015). In addition, the intensity of different biotic and abiotic interactions varies spatially, resulting in divergent selection and maintaining the variability of floral traits that influence these interactions (Mitchell-Olds et al., 2007; Ågren et al., 2013; Vaidya et al., 2018). The variability of floral traits may also result from indirect selection, imposed by either biotic or abiotic agents, on correlated vegetative traits (Simms and Bucher, 1996; Strauss and Whittall, 2006).

Floral color is a prevalent feature in the study of pollinator-mediated selection as it can offer a direct visual information to the flower visitors about the availability of rewards, and its variation at different levels (population, plant, flower) provides a basis for natural selection to occur (Kantsa et al., 2017; Wester and Lunau, 2017). Some pollinators can learn to associate specific flower colors with rewards, visiting preferentially those flowers (Grüter and Ratnieks, 2011; Grüter et al., 2011), which could cause directional selection on flower color. In fact, numerous studies have highlighted the role of pollinators in the evolution and maintenance of flower color diversity in plant clades (Kay and Sargent, 2009; van der Niet and Johnson, 2012), some of them in flower-color polymorphic species (Malerba and Nattero, 2012; Kellenberger et al., 2019). Although petal color may be affected by structural features of petal cells (Glover and Whitney, 2010; Airoldi et al., 2019; van der Kooi et al., 2019), it mainly results from the accumulation of pigments (Davies, 2000; Tanaka et al., 2008). Flavonoids are the principal source of flower color (Tanaka et al., 2008; Narbona et al., 2018), and they are related not only to pollinator attraction but also to defense against herbivory or pathogens or to protection from environmental stressors, such as UV-radiation, drought, temperature, or salinity (Chalker-Scott, 1999; Winkel-Shirley, 2002; Manetas, 2006; Gould et al., 2009; Lev-Yadun and Gould, 2009; Qi et al., 2011; Schaefer and Ruxton, 2011; del Valle et al., 2015; Chin et al., 2016; Davies et al., 2018). Therefore, flower color can act as a signal for mutualists and antagonists (Fineblum and Rausher, 1995; Irwin et al., 2003; Caruso et al., 2010; Ramos and Schiestl, 2019). In fact, some floral herbivores, such as Hemiptera (Farnier et al., 2014), Thysanoptera (Vernon and Gillespie, 1990; Gaum et al., 1994; Chyzik et al., 1995), Lepidoptera (Johnson et al., 2008), or pollen-feeding beetles (Giamoustaris and Mithen, 1996), discriminate among flower color variants resulting in higher attack and damage levels on some colors over others. Furthermore, it has been found that larvae from Lepidoptera species prefer to feed on flowers with low anthocyanin contents (Johnson et al., 2008) as these pigments reduce their growth (Johnson and Dowd, 2003; Johnson et al., 2008). Besides, due to those multiple roles of flavonoids, floral color may have diverse pleiotropic effects in other plant organs, through which selection, exerted by either biotic or abiotic factors, can indirectly act on flower color (Strauss and Whittall, 2006; Narbona et al., 2018).

Flower traits are frequently correlated (Mazer and Hultgård, 1993; Ishii and Morinaga, 2005). Diverse floral traits, such as floral display in *Lobularia* (Gómez, 2000), inflorescence production in *Hydrophyllum* (Wolfe, 1993), stigma-anther separation in *Ipomoea* (Sobrevila et al., 1989) and *Lysimachia* (Jiménez-López et al., 2020), nectar production in *Ipomopsis* (Meléndez-Ackerman and Campbell, 1998), or flower size in diverse crucifer species (Dukas and Shmida, 1989), have shown to be correlated to flower color. Given that biotic selective agents, such as pollinators and herbivores, also respond to other features apart from flower color, it is necessary to consider those correlations when studying selection on flower color (Kingsolver and Diamond, 2011).

Floral color has been mostly viewed by evolutionary biologists as a qualitative trait (Harder and Johnson, 2009), and studies assessing selection on that floral feature as a continuous trait are not frequent (but see Renoult et al., 2013; Sletvold et al., 2016). In shore campion (*Silene littorea* Brot.), flower color shows a genetically determined continuous variation from dark pink to white, which so far has been analyzed considering the dark pink, light pink, and white categories for simplicity, with frequency distribution of colors differing drastically among populations (Casimiro-Soriguer et al., 2016; Del Valle et al., 2019). Although *S. littorea* is self-compatible, it needs pollinator visitation to achieve complete seed production per fruit (Casimiro-Soriguer et al., 2015; Buide et al., 2020), and pollinators can thus be agents of phenotypic selection on flower color. Bees and butterflies were the main pollinators of *S. littorea* and they preferentially visited pink vs. white flowers (Buide et al., 2020). Since some pollinators, such as bees, can differentiate among flowers that differ subtly in color (Garcia et al., 2017), it is worth considering the whole variation from pink to white and the effect of such color variation on fitness. In addition, this species showed among-year variation in florivory, and white-flowered plants showed higher total levels of florivory (Buide et al., 2020). Moreover, the genus *Silene* usually exhibits a close relationship with the genus *Hadena*, which oviposits in ovaries and the larvae feed on the seeds (e.g. Koch, 1984; Giménez-Benavides et al., 2007; Kula et al., 2013); and in particular, *Hadena sancta* was found in *S. littorea* (Prieto-Benítez et al., 2017). In *S. littorea*, pigments responsible for pink petal coloration are anthocyanins, specifically cyanidin derivatives, which may also accumulate in sepals (Del Valle et al., 2019); their concentrations in corolla and calyx show wide variation and do not usually correlate (del Valle et al., 2015). The pink intensity of petals depends on their anthocyanin concentration (Casimiro-Soriguer et al., 2016); likewise, calyx color varies from green to dark red depending on anthocyanin accumulation (del Valle et al., 2015). That variability in pigment concentration, and thus in color, in petals and calyces could be involved in both pollinator and herbivore preferences. Hence, *S. littorea*, as a study model, gives an excellent opportunity to assess spatial variation in selection regimes on quantitative variation of floral color.

Here, we assess natural selection on the quantitative variation in the flower color found in two nearby populations of *S. littorea*, taking into account correlations to other floral traits and herbivore pressures. We use selection differentials and gradients to quantify phenotypic selection on floral traits through both male and female fitness, and we also use structural equation modeling (SEM) to disentangle the effects of floral traits and herbivory on female fitness and thus to unravel the ecological causes of selection. The simultaneous use of those analyses is recommended when assessing selection on quantitative traits and dealing with multiple causal scenarios (Conner et al., 1996; Gómez, 2000; Scheiner et al., 2000). Specifically, we raise the following questions: (1) Does natural selection operate on continuous variation of floral color? (2) Does selection on floral color act directly or indirectly through other floral traits? (3) Do herbivores affect selection on flower color? and (4) Is there any variation in selection regime between populations?

## Materials and Methods

### Study System

**Figure 1:**
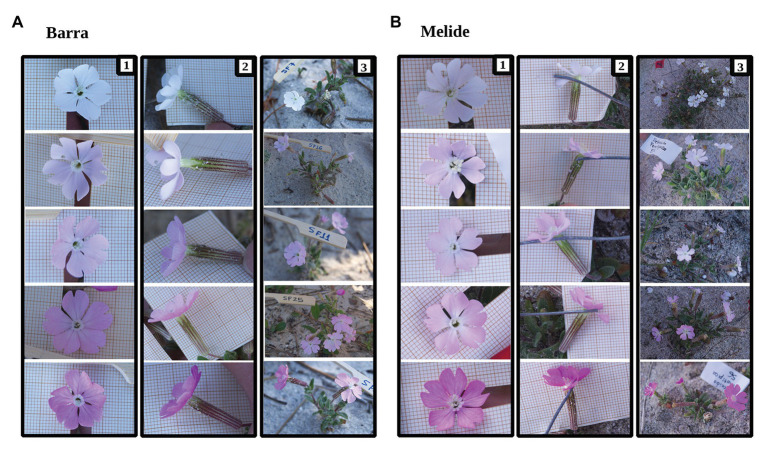
Plants of *Silene littorea* from Barra and Melide populations showing corolla color variation from dark pink to white (**A1**,**B1**), anthocyanin accumulation in calyces (i.e., redness; **A2**,**B2**), and the whole plant (**A3**,**B3**).

*Silene littorea* (Caryophyllaceae) is an annual plant distributed throughout the coast of the Iberian Peninsula and in the Northwest of Morocco (Talavera, 1990). It blooms between March and June and produces from only three to nearly 300 flowers per plant (Casimiro-Soriguer et al., 2013). It is a gynodioecious-gynomonoecious species with three sexual phenotypes: female plants, hermaphrodite plants, and gynomonoecious plants, with both female and hermaphrodite flowers (Guitián and Medrano, 2000; Casimiro-Soriguer et al., 2013). The relative frequencies of those sexual phenotypes are very variable among populations of Spain, female plants being scarce or absent in all reported populations and hermaphrodite plants being more frequent in populations of Northwest Spain (Guitián and Medrano, 2000; Casimiro-Soriguer et al., 2013). Hermaphrodite flowers are protandrous with 10 stamens that open sequentially mostly before the receptivity of the stigma, but some overlaps between sexual phases exist (Casimiro-Soriguer et al., 2013). *Silene littorea* is a self-compatible species, with incomplete protandry that allows some levels of autonomous selfing (Casimiro-Soriguer et al., 2015; Buide et al., 2020). Most populations of *S. littorea* consist exclusively of plants with pink flowers, while a few populations in the northwest of Spain also include white-flowered plants in proportions that differ across populations but are maintained over years (Casimiro-Soriguer et al., 2016; Del Valle et al., 2019). These populations include a continuous variation from dark-pink- to white-flowered plants, through a gradation of pinks (Figure 1). There is no information about the selective regimes operating to maintain flower color variability among plants in these populations.

This study was carried out during the spring season of 2016 in two wild populations in north-western Spain, Barra (42°15'42.20''N, 8°51'10.49''W) and Melide (42°15'5.18''N, 8°51'58.64''W), only separated by 2.6 km. Barra has maintained different proportions of white-flowered plants ranging from 14 to 20% through 3 consecutive years recorded (Del Valle et al., 2019). In the studied year, the proportions of white-flowered plants were 20% in Barra and 65% in Melide.

### Floral Traits

In this study, we have included those floral characters involved in attracting pollinators, such as flower color, flower size and floral display, on the one hand. Since some Lepidoptera oviposit in the ovary though the calyx and feed on the ovules and seeds of *Silene* (see above), we have considered, on the other hand, the color and size of the calyx and the number of ovules as factors that can affect the impact of herbivory. The number of flowers per plant was also included as it can have a direct relationship with the total fitness of the plant. Corolla size (measured as total area) and color, calyx length and color, ovules per flower, floral display, and total number of flowers per plant were recorded in 46–49 plants in Barra and 42–50 in Melide. Initially, 50 plants were tagged in each population, but in the end, there were some missing data for different traits. To avoid bias in phenotypic selection analyses due to different abundances of flower colors between populations, plants sampled in each population were evenly distributed along the corolla color gradient, from dark pink to white. In contrast, gender was not taken into account when sampling those plants, that is, plants were selected at random with respect to that feature although their gender expression was assessed when estimating flower production (see below). To measure color and size traits, a flower per plant was photographed in both lateral and zenith views with a digital camera (SONY SLT A65V) at the flowering peak; lateral pictures were used to measure calyx traits and zenith ones to characterize corolla traits. Each picture included a scale so that flower size was characterized, and the software ImageJ (version 1.52a, National Institutes of Health, USA) was used to measure corolla area and calyx length. Corolla and calyx colors were assessed on those pictures following the method described by Del Valle et al. (2018), which uses the output data from digital images to calculate indices that correlate to anthocyanin concentrations. Briefly, camera settings were manually adjusted for pictures: lens aperture F/5.6, ISO 100, white balance fixed at 4,500 K, and the integration time was changed depending on the specific daylight conditions (1/30 to 1/100). Pictures were taken in Sony Alpha Raw format (RAW) and flowers were photographed along with a Color-Checker Passport (X-Rite Inc., Grand Rapids, MI). Photographs were normalized and linearized using “Image Calibrations and Analysis Toolbox,” a plug-in module for ImageJ (Troscianko and Stevens, 2015), using two gray standards of the Color-Checker: Neutral 3.5 and Neutral 8. From each image, we selected a specific area of either corolla or calyx to be analyzed and extracted its mean pixel values of the red (R), green (G), and blue (B) channels. Then, anthocyanin concentration was estimated by the R:G ratio, that is, the mean pixel value of the red channel divided by that of the green channel, which is one of the indices that better correlates to anthocyanin concentration and color based on reflectance spectra in petals and calyxes of *S. littorea* (del Valle et al., 2018).

For each plant, ripe seeds and aborted ovules were counted in three unopened fruits; then, the number of ovules per flower was obtained by adding ripe seeds and aborted ovules. The floral display of each plant was obtained by counting all open flowers 1 day at the peak of flowering. Lastly, the total number of flowers produced per plant was estimated by counting weekly all open flowers along the flowering period, each flower being marked to avoid recounting it, and female and hermaphrodite flowers being recorded separately.

### Herbivory

Herbivory was assessed, as both corolla and ovary predation, for 49–50 of the plants used to measure floral traits, in each population. The percentage of corolla area predated was calculated on the same photographs used to measure the corolla size by using ImageJ software. For each plant, ovary predation was assessed by counting all ovaries showing chewing damage, and from that number, the proportion of predated ovaries, relative to total ovaries, was calculated.

### Fitness Estimates

In each population, most sampled plants were hermaphrodites and only a few were gynomonoecious. In *S. littorea*, female fitness has been reported to be independent from flower gender (Guitián and Medrano, 2000; Casimiro-Soriguer et al., 2013). Thus, for gynomonoecious plants, both female and hermaphroditic flowers were used to estimate female fitness and only hermaphroditic flowers to assess male fitness. For hermaphrodite plants, all the flowers were used to estimate both female and male fitness.

Female fitness was assessed in each population for 42–50 of the plants used to measure floral traits. For each plant, fruit production was assessed by counting all fruits produced and the fruit-set (proportion of flowers setting fruits) was estimated by relating that the number of fruits to that of total flowers produced. To estimate seed production per fruit and seed-set (proportion of ovules becoming seeds), both ripe seeds and aborted ovules were counted in three unopened fruits per plant. Lastly, the total female fitness were estimated for each plant as its total seed production, that is, as the product of its mean number of ripe seeds per fruit with its total fruit production.

Male fitness was assessed for a subsample of the plants used to measure floral traits (20 plants in Barra and 22 in Melide). The number of dispersed pollen grains was used as a proxy for male fitness (Snow and Lewis, 1993; Holland et al., 2004; Arista and Ortiz, 2007), estimating the number of pollen grains that remains in the anther after pollination. From each plant, one or two anthers per flower were collected just before their abscission and fixed in 70% ethanol. To count the number of undispersed pollen grains, the remaining pollen of each anther was dispersed in 1.5 ml of soapy distilled water. For each sample, the exact number of pollen grains was counted in 10 subsamples of 10 μl under a microscope. From this mean, the total number of undispersed pollen grains was determined. Then, for each flower, the number of dispersed pollens was estimated by subtracting the undispersed pollen grains from the mean number of pollen grains produced per flower of each population. Pollen production per flower from these populations had been previously obtained (21,711.33 ± 620.28; *n* = 30 in Barra and 18,192.3 ± 561.4; *n* = 42 in Melide; *t* = 4.21, *df* = 65.08, *p* < 0.00001). Lastly, the total male fitness for each plant was estimated as the product of its number of pollen grains dispersed per flower by its number of hermaphrodite flowers.

### Statistical Analysis

Data exploration was performed before analysis: normality was checked by the Shapiro-Wilk test using R package “Stats” (v3.6.2), and variance homogeneity was checked by the Levene test using R package “Car” (v3.0-8; Fox and Weisberg, 2019). Then, differences in floral traits, fitness, and herbivory between populations were tested using the Wilcoxon rank-sum test for all variables except for the calyx color index, the number of ovules per plant, the seed-set, and the number of seed per fruit, the only parametric ones, where *t*-test was used; both tests were performed with R package “Stats” (v3.6.2). Pairwise relationships among corolla color, floral display, and the number of flowers per plant were tested through Pearson correlations using the R package “Psych” (v1.9.12.31; Revelle, 2020). Before analyses, all variables were centered for each population to a zero mean with R package “Caret” (v6.0-86; Kuhn et al., 2016).

To explore whether the studied floral traits were under selection, we estimated selection opportunities, differentials, and gradients (Lande and Arnold, 1983; Arnold and Wade, 1984a,b). All those coefficients were estimated through both total male and total female fitness and separately for each population. We used the number of pollen grains dispersed and the number of seeds produced per plant as absolute measures of total male and female fitness, respectively. In order to better understand selection regimes in each studied population, selection differentials and gradients were also estimated through each of the partial components of fitness considered. Before estimating selection coefficients, all fitness measures were relativized (absolute value of each plant divided by the mean value in the population). Moreover, to calculate selection coefficients, each floral trait was standardized for each population to a zero mean and unit variance. We calculated selection opportunities as the variances in either total male or total female relative fitness; those coefficients measure the constraint on the evolutionary response imposed by fitness variability (Crow, 1958; O’Donald, 1970; Wade, 1979; Wade and Arnold, 1980; Arnold and Wade, 1984a,b; Moorad and Wade, 2013). To check for differences in selection opportunities between populations, we used R package “Car” (v3.0-8; Fox and Weisberg, 2019) to perform the Brown-Forsythe test, which is a robust test to compare variances of non-parametric data. We used R package “pbdDMAT” (v0.5-1; Schmidt et al., 2012) to estimate standardized selection differential (S') for each trait as the covariance between the standardized trait and either total male or total female relative fitness or any of the partial components of fitness considered; this coefficient measures the total selection acting on each trait, including both direct selection on the focal trait and indirect selection though correlated traits (Lande and Arnold, 1983). Lastly, we used multiple regression analyses to estimate standardized selection gradients, which indicate the force and direction of selection acting directly on each trait (Lande and Arnold, 1983). We used R package “Stats” (v3.6.2) to perform linear regressions of either total male or total female relative fitness or any of the partial components of fitness considered on all standardized traits; partial-regression coefficients (*β*') are standardized linear selection gradients representing the magnitude and direction of directional selection (Lande and Arnold, 1983).

To investigate complex multivariate causal models of plant phenotypic traits and herbivores on total female fitness, we used SEM, specifically path analysis (Grace, 2006; Rosseel, 2012). This analysis technique is appropriate to test our hypothesis, since it allows the simultaneous evaluation of the effects of multiple pathways between variables by comparing observed and expected covariance matrices. Our hypothesis was tested in models using unstandardized data (Grace, 2006), except for total female fitness, which was divided by 1,000 to reduce the effect of a larger variance. Corolla color was defined as an independent variable, and calyx color, total number of flowers per plant, percentage of corolla area predated, proportion of ovaries predated per plant, and female fitness (estimated as total seeds produced per plant) were defined as dependent variables. To choose the model that best fit our data, we first used an alternative model evaluation approach (Hershberger et al., 2003; Grace, 2006), by comparing near-saturated models (i.e., models with only one and two degrees of freedom) differing in the order of unconnected variables (see the saturated model in Supplementary Figure S1). This process generated 102 different models for each population (Supplementary Table S1). We used this method because, *a priori*, all paths connecting the variables could be biologically possible and relevant (see for instance Cariveau et al., 2004; Swope and Parker, 2012; Carlson and Holsinger, 2013). The best-fitting model was chosen based on the lowest Akaike information criterion (AIC) values (Grace, 2006), which also were consistent with the Bayesian information criterion (BIC). Second, we performed the nested model comparisons using the best-fitting model as the baseline model (Grace, 2006). Model trimming was achieved by eliminating pathways with the lowest path coefficients, non-statistically supported. Comparisons between baseline model and reduced models were tested with Chi-square tests based on the differences of the two models’ adjustment (Pugesek, 2003; Grace, 2006). However, differences between Chi-squares do not allow discrimination among the nested models (values of *p* < 0.4; Supplementary Table S2) and, thereby, we again applied the lowest AIC and BIC criteria.

Path analyses were performed using the “sem” function from R package “Lavaan” version 0.6-6 (Rosseel, 2012, 2020) with the maximum likelihood estimation of parameters. Because the data were not the multivariate normal distribution, we used bootstrap-based estimates of standard errors (*n* = 1,000; Grace, 2006; Rosseel, 2020). All statistical packages used throughout this article were implemented in R v.3.6.3 (R Core Team, 2020).

## Results

### Variability in Floral Traits, Herbivory, and Fitness

As expected, the studied samples of both populations did not differ in corolla color (Table 1) because the previous selection of plants to include the overall color variation in both populations (see methods). Plants from Melide had smaller flowers with fewer ovules and darker calyces than those from Barra (Table 1). For all other traits, no differences were found between populations. Floral display and the total number of flowers per plant showed the highest variation among all the measured floral traits (Table 1), and these two traits were correlated in both populations (Barra: Pearson coefficient = 0.49, *p* < 0.001, *n* = 74; Melide:Pearson coefficient = 0.83, *p* < 0.001, *n* = 50).

**Table 1:** Floral traits and herbivory and fitness estimates for Barra and Melide populations of *Silene littorea*.

	Barra					Melide					Between population comparisons
	Mean ± SD	Min	Max	N	CV %	Mean ± SD	Min	Max	N	CV %	
**Floral traits**											
Corolla color	1.29 ± 0.18	1.02	1.68	49	14.17	1.25 ± 0.17	1.03	1.60	50	13.23	*W* = 1,371, NS
Calyx color	1.35 ± 0.10	1.11	1.63	47	7.51	1.43 ± 0.10	1.17	1.69	50	7.18	*t* = −3.716, *p* < 0.001
Flower size (cm^2^)	2.46 ± 0.50	1.38	3.93	49	20.38	1.85 ± 0.41	1.29	3.26	50	22.25	*W* = 2,040, *p* < 0.0001
Calyx length (cm)	1.71 ± 0.26	0.92	2.42	46	14.96	1.78 ± 0.16	1.44	2.25	50	9.24	*W* = 973, NS
Ovules per flower	66.85 ± 10.00	38.67	85.00	46	14.96	62.20 ± 8.07	40.00	77.33	42	12.98	*t* = 2.4092, *p* < 0.05
Floral display	2.51 ± 1.52	1.00	7.00	49	60.37	3.02 ± 2.22	1.00	13.00	50	73.43	*W* = 1,088, NS
Flowers per plant	17.41 ± 11.40	1.00	51.00	49	65.49	17.12 ± 12.44	2.00	53.00	50	72.67	*W* = 12,81.5, NS
**Herbivory estimates**											
Predated corolla area (%)	0.49 ± 1.04	0.00	5.52	49	213.82	1.00 ± 1.56	0.00	6.10	50	155.93	*W* = 986.5, NS
Ovaries predated per plant (%)	0.14 ± 0.15	0.00	0.62	49	105.31	0.18 ± 0.16	0.00	0.67	50	90.28	*W* = 1,054.5, NS
Ovaries predated per plant	2.71 ± 3.21	0.00	14.00	49	118.43	3.66 ± 4.70	0.00	24.00	50	128.34	*W* = 1,101.5, NS
**Fitness estimates**											
Fruit-set	0.32 ± 0.16	0.00	0.78	49	48.72	0.24 ± 0.15	0.00	0.75	50	62.99	*W* = 1,643.5, *p* < 0.005
Seed-set	0.65 ± 0.19	0.16	0.95	46	30.07	0.51 ± 0.22	0.03	0.91	42	44.21	*t* = 3.1574, *p* < 0.005
Fruits per plant	5.78 ± 4.33	0.00	19.00	49	75.00	3.86 ± 2.93	0.00	15.00	50	76.03	*W* = 1,606.5, *p* < 0.01
Seeds per fruit	43.04 ± 14.00	9.50	72.67	46	32.53	30.79 ± 14.99	2.00	64.33	43	48.69	*t* = 3.9771, *p* < 0.0005
Seeds per plant	272.69 ± 251.55	0.00	1,095.67	49	92.25	126.37 ± 139.11	0.00	710.00	47	110.08	*W* = 1,609.5, *p* < 0.001
Dispersed pollen grains per flower	17,856.10 ± 5,076.38	602.33	21,252.33	20	28.43	16,222.72 ± 3,206.86	4,833.45	18,258.45	22	19.77	*W* = 331, *p* < 0.01
Dispersed pollen grains per plant	427,694.97 ± 258,064.62	12,649.00	985,552.33	20	60.34	355,934.71 ± 251,876.43	64,633.81	919,202.98	22	70.76	W = 262, NS

Statistical comparisons between populations is showed. Corolla and calyx color index were measured as R:G ratio (see section “Materials and Methods”).

The percentage of corolla area predated was similar in both populations. Likewise, no differences between populations were found on number and percentage of predated ovaries (Table 1). No direct observation was made on the activity of predation agents, but *Hadena* larvae and Curculionidae eggs were found in flowers and fruits examined to characterize floral traits and to assess fitness.

All estimates of female fitness differed between populations. Fruit-set, seed-set, and seeds per fruit were markedly higher in Barra (Table 1), but the most marked difference affected seed production per plant, which was more than twice in Barra than in Melide, even though the total flowers per plant did not differ between populations (Table 1). In contrast, differences between populations in male fitness were much less marked, and although the two estimates considered showed a trend to be higher in Barra, only the difference in the number of pollen grains dispersed per flower was significant (Table 1).

### Phenotypic Selection on Flower Color and Other Flower Traits

Selection opportunities did not differ between populations either for total female fitness (0.85 in Barra and 1.21 in Melide; *F*_1_,_94_ = 0.06, *p* = 0.81, Brown-Forsythe test) or total male fitness (0.36 in Barra and 0.50 in Melide; *F*_1_,_40_ = 0.54, *p* = 0.47, Brown-Forsythe test). In contrast, patterns of selection varied between the two populations (Table 2).

**Table 2:** Phenotypic selection coefficients of floral traits of *S. littorea* through partial components of fitness, either female (fruit-set, fruits per plant, seed-set, and seeds per fruit) or male (dispersed pollen grains per flower), and through total female (seeds per plant) and total male (dispersed pollen grains per plant) fitness in Barra and Melide populations.

Population	Trait	Fruit-set		Fruits/plant		Seed-set		Seeds/fruit		Seeds/plant		Dispersed grains/flower		Dispersed grains/plant	
		S'	β'(SE)	S'	β'(SE)	S'	β'(SE)	S'	β'(SE)	S'	β'(SE)	S'	β'(SE)	S'	β'(SE)
Barra	Corolla color	−0.04	−0.07 (0.08)	0.16	−0.04 (0.07)	−0.06	−0.11w^*^ (0.05)	−0.05	−0.11^*^ (0.05)	0.15	−0.11 (0.1)	0.00	0 (0.12)	0.19^*^	0.01 (0.1)
	Calyx color	0.02	0.08 (0.07)	0.26^**^	0.15^*^ (0.07)	−0.02	−0.01 (0.05)	0.00	0 (0.05)	0.31^**^	0.19 (0.1)	0.07	0.08 (0.11)	0.30^***^	0.08 (0.09)
	Corolla size (cm^2^)	−0.06	−0.08 (0.07)	0.03	−0.06 (−0.07)	−0.05	−0.09 (0.05)	−0.05	−0.10^*^ (0.05)	0.00	−0.14 (0.09)	0.04	0.02 (0.1)	0.12	0 (0.08)
	Calyx length (cm)	0.12	0.08 (0.07)	0.06	0.07 (0.07)	0.00	−0.03 (0.05)	−0.02	−0.03 (0.05)	0.07	0.07 (0.09)	0.04	0.02 (0.11)	−0.05	0.05 (0.1)
	Ovules per flower	0.03	0.1 (0.09)	0.34^***^	0.11 (0.09)	−0.04	−0.04 (0.06)	0.12^**^	0.12^*^ (0.06)	0.42^***^	0.21 (0.12)	0.06	0.09 (0.15)	0.26^***^	0.06 (0.13)
	Floral display	0.02	0 (0.1)	0.48^***^	0.02 (0.1)	−0.02	−0.1 (0.07)	0.07	−0.1 (0.07)	0.54^***^	−0.09 (0.13)	0.02	−0.07 (0.13)	0.38^***^	−0.04 (0.11)
	Flowers per plant	0.04	−0.11 (0.1)	0.62^***^	0.41^***^ (0.1)	0.03	0.15^*^ (0.07)	0.11^*^	0.15^*^ (−0.07)	0.71^***^	0.51^***^ (0.13)	0.04	−0.02 (0.13)	0.56^***^	0.44^**^ (0.11)
Melide	Corolla color	−0.01	0 (0.11)	−0.24^*^	−0.02 (0.1)	−0.01	−0.02 (0.09)	−0.02	−0.01 (0.09)	−0.33*	−0.1 (0.17)	0.06^*^	0.15 (0.07)	−0.22^*^	0.15 (0.1)
	Calyx color	0.02	0.04 (0.1)	−0.06	−0.03 (0.09)	−0.02	−0.03 (0.08)	−0.01	−0.02 (0.08)	−0.06	−0.04 (0.14)	−0.03	0.01 (0.06)	−0.19^*^	−0.04 (0.08)
	Corolla size (cm^2^)	−0.07	−0.15 (0.11)	0.03	−0.08 (0.1)	0.05	0.07 (0.09)	0.08	0.06 (0.09)	0.07	−0.04 (0.16)	−0.02	−0.13 (0.07)	0.00	−0.26^*^ (0.1)
	Calyx length (cm)	0.10	0.08 (0.11)	−0.06	0 (0.1)	−0.04	−0.06 (0.09)	0.02	−0.06 (0.09)	−0.03	0.03 (0.17)	−0.03	0.08 (0.07)	−0.20	0.12 (0.1)
	Ovules per flower	−0.13	−0.09 (0.11)	0.11	−0.07 (0.1)	−0.01	0 (0.09)	0.12	0.14 (0.09)	0.23	−0.01 (0.16)	−0.01	−0.11 (0.08)	0.24^**^	−0.14 (0.1)
	Floral display	−0.11	0.14 (−0.2)	0.44^***^	−0.05 (0.18)	0.02	−0.07 (0.16)	0.06	−0.07 (0.16)	0.56^***^	−0.28 (0.3)	0.02	0.3 (0.14)	0.72^***^	0.53^*^ (0.19)
	Flowers per plant	−0.11	−0.26 (0.17)	0.58^***^	0.61^***^ (0.15)	0.02	0.05 (0.14)	0.09	0.06 (0.14)	0.78^***^	0.93^***^ (0.26)	−0.03	−0.1 (0.1)	0.74^***^	0.41^*^ (0.13)

S', standardized selection differentials; β', standardized linear gradients of selection.

* Values in bold are significant: *p* < 0.05.

** Values in bold are significant: *p* < 0.01.

*** Values in bold are significant: *p* < 0.001.

In Barra population, four traits were under both indirect (S' significant) and direct (*β*' significant) selection by one or more fitness measures, one trait was only under indirect selection, and another one was only under direct selection (Table 2). Corolla color was under indirect positive selection *via* dispersed grains/plant and under direct negative selection *via* seed-set and seeds/fruit. Calyx color was under indirect positive selection *via* fruits/plant, seeds/plant and dispersed grains/plant and under direct positive selection *via* fruits/plant. Corolla size was under direct negative selection *via* seeds/fruit. The number of ovules per flower was under indirect positive selection *via* fruits/plant, seeds/fruit, seeds/plant, and dispersed grains per plant and under direct positive selection *via* seeds/fruit. Floral display was under indirect positive selection *via* fruits/plant, seeds/plant, and dispersed grains/plant. Flowers per plant was under indirect positive selection *via* fruits/plant, seeds/fruit, seeds/plant, and dispersed grains/plant and under direct positive selection *via* fruits/plant, seed-set, seeds/fruit, seeds/plant, and dispersed grains/plant.

In Melide population, two traits were under both indirect and direct selection, three traits were only under indirect selection and one was only under direct selection (Table 2). Corolla color was under indirect negative selection *via* fruits/plant, seeds/plant and dispersed grains/plant; and under indirect positive selection *via* dispersed grains/flower. Calyx color was under indirect negative selection *via* dispersed grains/plant. Corolla size was under direct negative selection *via* dispersed grains/plant. Number of ovules per flower was under indirect positive selection *via* dispersed grains/plant. Floral display was under indirect positive selection *via* fruits/plant, seeds/plant and dispersed grains/plant; and under direct positive selection *via* dispersed grains/plant. Flowers per plant were under indirect and direct positive selection *via* fruits/plant, seeds/plant and dispersed grains/plant.

### Effects of Floral Traits and Herbivores on Total Female Fitness

**Figure 2:**
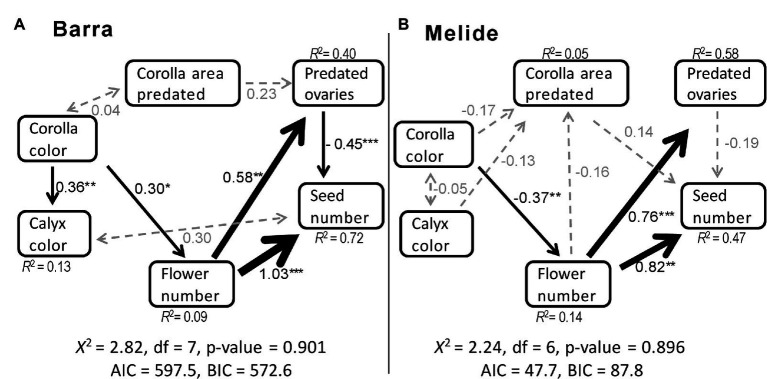
Results of the best structural equation model for the effects of corolla color, calyx color, percentage of area of corolla predated, number of predated ovaries per flower, and number of flower per plant on seeds produced per plant in **(A)** Barra and **(B)** Melide populations of *S. littorea*. Black single-pointed arrows represent causal relationships with significant standardized path coefficients (^*^p < 0.05, ^**^p < 0.01, ^***^p < 0.001), gray single-pointed arrows with dashed lines represent causal relationships with non-significant path coefficients, and gray double-pointed arrows represent correlations. Arrow-width indicates the relative magnitude of the path coefficient. R2 values are the percentage of variation explained by the causal variables. *X*^2^, Chi-square test; AIC, Akaike information criterion; BCI, Bayesian information criterion.

After the alternative model evaluation and subsequent nested model comparisons (Supplementary Table S1), the models with the best fit for Barra and Melide populations are shown in Figure 2. In Barra, the model retains five significant casual pathways, one non-significant casual path and two unresolved causal relationships, whereas in Melide the model retains three significant casual pathways, five non-significant casual pathways and one unresolved causal relationships (Supplementary Table S2; Figure 2).

According to the model selected for Barra, the number of flowers per plant had the highest significant positive effect (i.e., path coefficient) on total female fitness (seeds per plant; standardized path coefficient = 1.03, Figure 2A). The only significant negative effect was the number of predated ovaries per plant on total female fitness (standardized path coefficient = −0.45). In addition, the number of predated ovaries was positively affected by flower number (standardized path coefficients = 0.58), and both calyx color and flower number were positively influenced by corolla color (standardized path coefficients = 0.36 and 0.30, respectively). As a result, corolla color showed a significant positive indirect effect on female fitness *via* the number of flowers (Estimate = 0.418, Z = 2.28, *p* = 0.022; Supplementary Table S2; Figure 2A).

In Melide, the number of flowers per plant was also the trait with the highest significant effect on female fitness, but the number of flowers also had important influence on ovary predation (standardized path coefficients = 0.82 and 0.76, respectively, Figure 2B). In this population, calyx color was not significantly affected by corolla color and female fitness was not significantly affected by predated ovaries per flower. Corolla color showed a significant negative effect on flower number (standardized path coefficient = −0.37), meaning that plants with lighter corollas had a higher number of flowers per plant. In that way, corolla color had a marginally significant negative indirect effect on female fitness *via* number of flower (Estimate = −0.257, *Z* = −1.85, *p* = 0.064; Supplementary Table S2; Figure 2B).

## Discussion

In this research, we found among-population differences in floral traits and in patterns of phenotypic selection in two nearby populations of *S. littorea* with a wide continuous variation in petal color from dark pink to white. Patterns of phenotypic selection varied through either male or female fitness although two traits were subjected to consistent and marked selective pressures in both populations: floral display and total number of flowers per plant.

In both populations, the number of flowers per plant was subjected to both direct and indirect selection through both total male and total female fitness, and path analysis confirmed a direct effect of floral production on total female fitness. The association between flower production and female fitness is quite common, and it has been found in a wide variety of plants (e.g. Herrera, 1993; Gómez, 2000; Harder and Johnson, 2009); however, the impact of flower production on male fitness has been more rarely reported (but see Maad, 2000). Total flower production and floral display showed a strong correlation in both populations, a fact that is usual (Worley and Barrett, 2000; Sletvold et al., 2016), although floral display may be more constant among species than the total flower number (Kudo and Harder, 2005). Floral display is one of the most important determinants of a plant’s visual display and pollinators usually select plants with large floral displays (e.g., Eckhart, 1991; Thompson, 2001; Mitchell et al., 2004; Carlson and Holsinger, 2013). However, in our study, selection on floral display generally acted indirectly.

Since plants with large floral display and high flower production were strongly selected in both studied populations, an increase of these kinds of plants would be expected. However, these two traits showed the highest coefficients of variation among the studied traits, and in former studies, in these populations, flower production had also been reported to be highly variable (Casimiro-Soriguer et al., 2013). Other studies have reported a moderate heritability of flower production (Campbell and Halama, 1993; Worley and Barrett, 2000), and in *S. littorea*, this trait has a genetic component and also a plastic response to light conditions (Buide et al., 2018). These facts could be responsible for the maintenance of variability in this trait despite selective pressures against plants with fewer flowers. Herbivory could also be limiting the evolutionary response at least in Barra, since in both populations the path analysis revealed a positive effect of flower production on ovary predation that, in turn, had a negative effect on the total female fitness but that the latest effect was not significant in Melide.

Flower color showed different patterns of association with flower production in the two studied populations. In Melide, white- or lighter-flowered plants had higher production of flowers, and they were indirectly selected through both total male and total female fitness. In fact, in that population, path analysis revealed no direct effect of corolla color on total female fitness but an indirect effect through flower production, that is, lighter-flowered plants produced more flowers, and as a consequence, more seeds. The strong effect of flower production on total female fitness in that population could be hiding the impact of pollinators as selective agents on flower color. The lower fruit-set and seed-set recorded in that population could indicate a deficient pollinator visitation. However, there was no relation between flower color and either fruit-set or seed-set; and thus, plants in that population seem to receive low pollinator visits irrespective of their color, which would rule out pollinators as selective agents of flower color. Then, since predation of either flowers or ovaries had no effect on total female fitness in Melide, the overall results suggest that the high proportion of white-flowered plants in this population is a direct result of their higher fecundity due to their highest flower production.

A contrasting selective scenario appears in Barra, where indirect positive selection on petal color was found through absolute male fitness, that is, darker-flowered plants dispersed more pollen grains. Similar to that found in Melide, the path analysis does not show a direct effect of corolla color on total female fitness but an indirect effect through flower production. However, in Barra, the higher flower production was found in plants with darker flowers that consequently produced more seeds. Previous studies had found autonomous selfing in *S. littorea*, although pollinator visitation was required to reach full seed-set (Casimiro-Soriguer et al., 2015; Buide et al., 2020). In Barra population, Buide et al. (2020) reported that pollinators preferably visited pink flowers, and white-flowered plants under free pollination reached fruit-set values similar to those obtained by autonomous selfing. Thus, the lower male and female fitness of white-flowered plants in Barra could be related to their lower pollinator attraction, but the lower fitness seems mainly due to their lower flower production. However, their ability to produce fruits autonomously helps to compensate the scarcity of pollinators and to maintain corolla color variation in this population.

In Barra, ovary predation had a significant negative impact on total female fitness. In other species, such as *Petunia* (Johnson et al., 2008) and *Raphanus sativus* (Strauss et al., 2004), herbivory rates are higher in the flower parts or plants with lower levels of anthocyanins, and are thus less protected against herbivores. However, the path analysis does not support these hypotheses, as herbivory affected similarly all color phenotypes in the studied year. However, we cannot discard a different effect among years because in the previous year, we had found higher levels of florivory in white-flowered plants than in pink-flowered plants (Buide et al., 2020). Thus, future studies should include among-year variation.

Our results, although obtained with a limited sampling, suggest that the prevalence of white-flowered plants in Melide and pink-flowered plants in Barra can be an indirect effect of correlations between corolla color and other floral traits. In other species, when white-flowered mutants arise, they usually disappear or remain in low frequency in populations due to a variety of factors: low pollinator visitation, higher herbivory rates, poor abiotic performance, or genetic drift (Rausher, 2008; Narbona et al., 2018). Most populations of *S. littorea* through the Iberian Peninsula fit this situation as they have exclusively pink-flowered individuals and white mutants that sporadically appear are lost (Del Valle et al., 2019). The presence of white-flowered plants that are maintained over time occurs only in a few populations (Del Valle et al., 2019) and they dominate only in Melide. The strong correlation between flower production and petal color seems to explain the maintenance and high frequency of white- or pink- flowered plants in different populations and the soundness of the path-models support that result. The correlation between flower color and flower display or flower production has been found in other polymorphic species. In some taxa, such as *Lobularia maritima* (Gómez, 2000), *Claitonia virginica* (Frey, 2004), or *Protea aurea* (Carlson and Holsinger, 2013), the less pigmented morphs show higher floral display but in others, such as *Hydrophyllum appendiculatum* (Wolfe, 1993) or *Phlox drummondii* (Levin and Brack, 1995), the reverse situation is found. Those contrasting correlations between flower color and flower production can be explained by different pleiotropic relationships (Armbruster, 2002). However, in *S. littorea*, the correlation between petal color and flower production is not easy to explain, because it differed among populations. Although abiotic conditions are seemingly similar in both populations, the fact that flowers in Melide are smaller and with fewer ovules and pollen grains could indicate a lower resource availability in that population. A possible explanation for the differences between the two populations could be an interactive effect between habitat characteristics; for instance, resource availability combined with biotic factors could result in the different correlations between flower number and color. In Barra, the higher pollinator visitation and lower florivory of pink flowers (Buide et al., 2020) could lead to a positive correlation between flower number and color, if one assumes that there is no resource limitation and that pink flowers are more costly due to pigment production (Drumm-Herrel and Mohr, 1985). In contrast, in Melide, the combination of resource limitation with a low prevalence of pollinators could result in the negative correlation between flower number and color, hence higher flower production in white-flowered plants. Anyway, a more thorough analysis of resources availability and biotic factors over years should be done in those populations to unravel that possible combined effect. Alternatively, between-population differences in floral correlations could also be explained as a founder effect (Forsman et al., 2012) or could be due to our limited sampling. Nevertheless, it is difficult to explain differences between populations without knowing the extent to which the relationship between flower color and number is genetically and/or environmentally controlled (Wennersten and Forsman, 2012).

The different patterns of selective regimes in the studied populations, if maintained over time, could have evolutionary consequences for the maintenance of color polymorphism in *S. littorea*. Given that this trait is genetically determined (Casimiro-Soriguer et al., 2016), an increase of white-flowered plants is expected in Melide while a decrease would be expected in Barra. Directional selection could lead to the loss of flower color polymorphism within populations, although inter-population variation in selection regime could maintain flower color variability in the species as well as autonomous selfing. Lastly, although flower color was not under direct selection by pollinators or herbivores in the studied year, among-year variation and other important traits as flower odor should be considered to support indirect selection on flower color in *S. littorea*.

## Data Availability Statement

The raw data supporting the conclusions of this article will be made available by the authors, without undue reservation.

## Author Contributions

EN and MB conceived the experiments. MB and NR-C carried out the sampling. NR-C performed the data analysis. NR-C, MA, EN, PO, and MB wrote the article. All authors contributed to the article and approved the submitted version.

### Conflict of Interest

The authors declare that the research was conducted in the absence of any commercial or financial relationships that could be construed as a potential conflict of interest.
