# Supplementary figures and images for: Indirect Selection on Flower Color in Silene littorea
Source: Front Plant Sci. 2020 Dec 23;11:588383. doi: 10.3389/fpls.2020.588383 (PMC7785944; doi:10.3389/fpls.2020.588383)

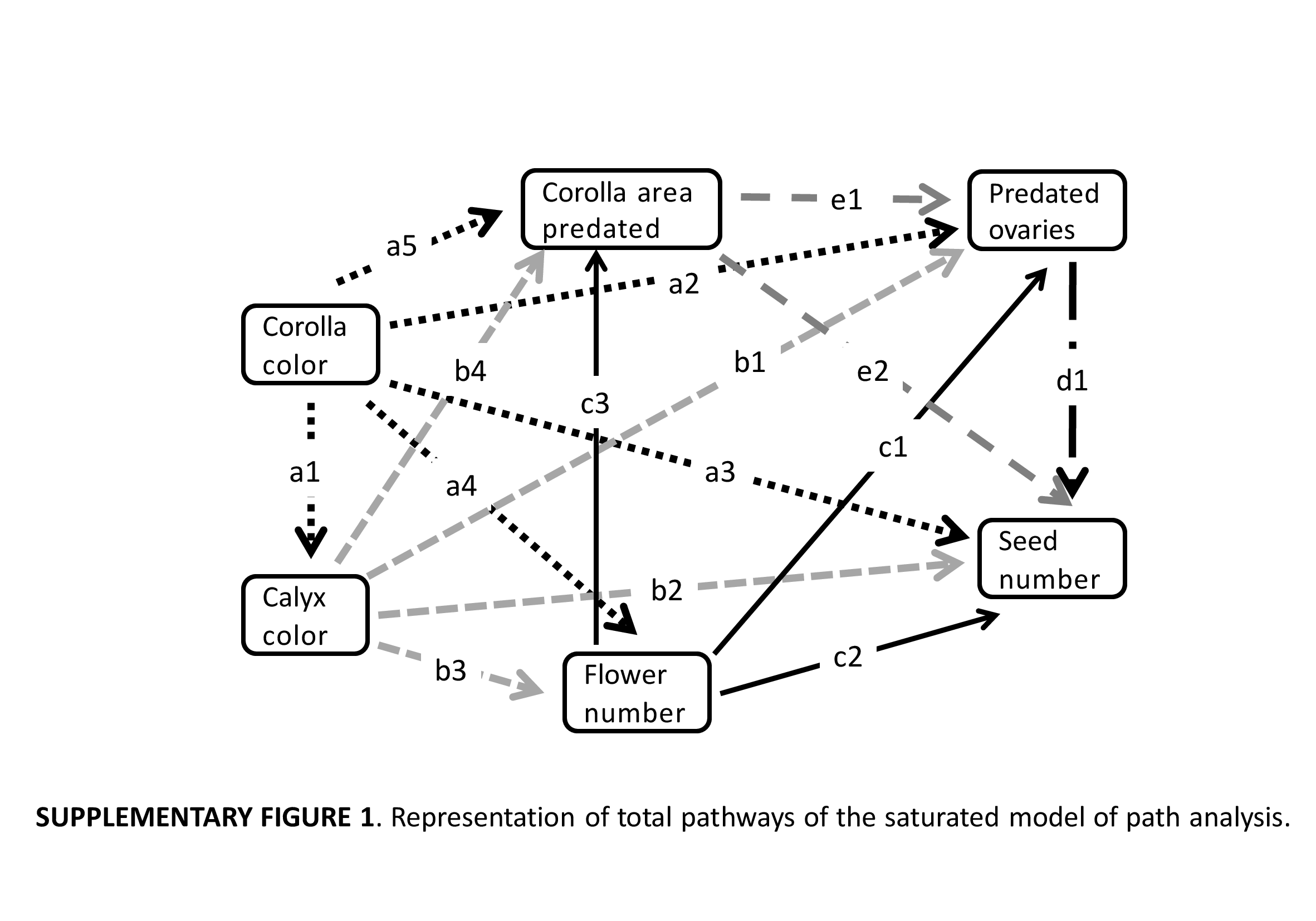

Supplement: Supplementary file 1 [file Image_1.tiff]
